# The biology of uterine sarcomas: A review and update

**DOI:** 10.3892/mco.2013.124

**Published:** 2013-05-20

**Authors:** HIROSHI KOBAYASHI, CHIAKI UEKURI, JURIA AKASAKA, FUMINORI ITO, AIKO SHIGEMITSU, NATSUKI KOIKE, HIROSHI SHIGETOMI

**Affiliations:** Department of Obstetrics and Gynecology, Nara Medical University, Nara 634-8522, Japan

**Keywords:** uterine sarcoma, carcinosarcoma, leiomyosarcoma, endometrial stromal sarcoma, tumor biology

## Abstract

Uterine sarcoma is a rare neoplasm, accounting for only 5% of uterine malignancies. The pathogenesis of uterine sarcoma remains largely unknown, although recent basic science and pre-clinical animal models have provided a better understanding of tumor biology. The aim of this study was to review the clinical features, imaging characteristics, genetic aberrations and therapeutic approaches in uterine sarcoma. This study reviewed the English-language literature on clinical and basic studies on uterine sarcoma. The common variants of uterine sarcoma are carcinosarcoma, leiomyosarcoma and endometrial stromal sarcoma (ESS). Genetic profiling efforts have identified amplification, overexpression and mutation, while the molecular mechanisms of tumorigenesis driven by these genomic and genetic aberrations have yet to be fully elucidated yet. Recent genome-wide studies have also identified complex chromosomal rearrangements as oncogenic mechanisms. The cell cycle regulators, p16 and p53, are frequently over-expressed and appear to be involved in key modifications of sarcomagenesis. Molecular-targeted therapy has now been evaluated in clinical trials for certain subtypes. In conclusion, aberrations of cell cycle control would be a critical step in the development of uterine sarcoma. This review has provided new areas of study targeting molecular and genetic pathways.

## Contents

1. Introduction
2. Literature search
3. Carcinosarcoma
4. Leiomyosarcoma
5. Smooth muscle tumor of uncertain malignant potential (STUMP)
6. Endometrial stromal sarcoma (ESS)
7. Adenosarcoma
8. Conclusion

## 1. Introduction

Uterine sarcoma accounts for 5% of all uterine malignancies (1). This tumor has been considered as highly aggressive, showing a poor prognosis. It mainly comprises the pathological subgroups of carcinosarcoma [formerly known as malignant mixed Müllerian tumor, (MMMT)] as the most common histology (50%), leiomyosarcoma (30%) and the remaining consists of endometrial stromal sarcoma (ESS) (10%). Each group has its own risk factors and clinical manifestations, including abnormal vaginal bleeding and pain, treatment response, prognosis and specific genetic aberrations.

This study is a review of the literature with the aim of understanding the natural history and clinical characteristics of uterine sarcomas, identifying specific cytogenetic aberrations and molecular genetic alterations and developing a treatment strategy. Identification of genomic and genetic alterations provides insights into their molecular biology, potential diagnostic markers, and possible therapeutic targets. In this review, the recent advances in the molecular biology of uterine sarcoma were investigated.

## 2. Literature search

A computerized literature search was performed to identify relevant studies written in English. MEDLINE updates were conducted on a monthly basis, and the abstracts were reviewed to identify manuscripts for full-text review. PubMed MEDLINE electronic databases (http://www.ncbi.nlm.nih.gov/sites/entrez) published until September 2012 were searched, combining the key words ‘uterine sarcoma’, ‘carcinosarcoma’, ‘leiomyosarcoma’, ‘endometrial stromal sarcoma’, ‘adenosarcoma’, ‘smooth muscle tumor of uncertain malignant potential’, ‘MR imaging’, ‘genome-wide’ and ‘clinical study’. Various combinations of the terms were used, depending on the database searched. Each gene was also linked to NCBI Entrez Gene pages (http://www.ncbi.nlm.nih.gov/sites/entrez). Additionally, references in each manuscript were searched to identify potentially missed studies.

## 3. Carcinosarcoma

### Clinical features

Carcinosarcoma, formerly referred to as MMMT, is the most common uterine sarcoma and accounts for ∼1/2 of uterine sarcomas and 2% of malignant uterine tumors (1). Carcinosarcoma is characterized by a combination of carcinomatous (epithelial) and sarcomatous (mesenchymal) elements derived from a single epithelial cell precursor or a stem cell. Carcinosarcoma is a tumor of monoclonal origin that develops phenotypic diversity (2). It is thought that the sarcomatous portions are derived from the carcinomatous elements. The carcinoma component may play an important role in aggressive biologic behavior and determines prognosis. Uterine carcinosarcoma appears at advanced ages. The age range at the time of diagnosis is 50–70 years. A prolonged exposure to tamoxifen and radiotherapy are two causal factors for the increased risk of carcinosarcoma.

### Imaging characteristics

Carcinosarcoma resembles a bulky polypoid mass that fills the endometrial cavity and often prolapses through the endocervical canal. The most prevalent ultrasonographic features are a mass with a heterogeneous appearance, increased vascularity and lacking internal calcifications. The tumor generally appears as areas of heterogeneous high intensity on T2-weighted magnetic resonance (MR) images and low intensity on T1-weighted images. Areas of elevated T1 signal are present in some lesions, which corresponded to hemorrhage and necrosis. Necrotic areas are hypointense on diffusion-weighted images and high apparent diffusion coefficient (ADC) values, which may be useful for the diagnosis of this disease. The lesions typically show a prolonged intense enhancement with greater heterogeneity which is the most common imaging feature on contrast-enhanced MR imaging.

### Genetic aberrations

Chromosomal amplification is not random and is closely associated with chromosomes 8q and 20q (42 and 70%) (Table I) (3). The two chromosomes contain oncogenes, c-myc (v-myc myelocytomatosis viral oncogene homolog; 8q24.12) and ZNF217 (zinc finger protein 217; 20q13.2), which are relevant to various types of tumor. In general, amplification of c-myc correlates with distant metastases and is a factor of poor prognosis. c-myc amplification was observed more often in the carcinomatous compared to the sarcomatous component. Thus, the proliferation and aggressiveness were associated with the presence of carcinomatous component. Furthermore, transcription factor ZNF217 overexpression was observed in the two tumor components and regulated by the hypoxia-inducible factors (HIFs) released by the hypoxic environment of the tumor. Findings of a recent study have demonstrated that ZNF217 is a biomarker of poor prognosis in numerous types of cancer (4).

Overexpression of specific genes, including transforming growth factor (TGF)-β, retinoblastoma (Rb), p53, HER-2 (also known as ERBB2, v-erb-b2 erythroblastic leukemia viral oncogene homolog 2), vascular endothelial growth factor (VEGF), estrogen receptor (ER), progesterone receptor (PR), cancer-testis-associated antigens (CATs), β-catenin, B-cell CLL/lymphoma 2 (BCL-2), cyclooxygenase (COX)-2, p16^INK4a^ (CDKN2A, cyclin-dependent kinase inhibitor 2A), phosphatase and tensin homolog (PTEN) and vimentin, has been detected in several cases (Table I). Positive immunostaining for steroid receptors (ERα and PR), cytokeratin, and EGFR was detected only in the carcinomatous component, whereas β-catenin, VEGF, BCL-2, COX-2, Rb and vimentin were immunoreactive in both components (5).

TGF-β has a distinct role in carcinosarcoma associated with the whole epithelial to mesenchymal transition (EMT) process, which may be responsible for the morphologically observed shift towards the mesenchymal character (5). Epithelial cells are potentially able to undergo EMT in response to TGF-β-smad3 signaling, as well as other stimuli such as changes in the Akt/β-catenin pathway, as well as alterations in Rb, Notch, Hedgehog and serine/threonine kinase glycogen synthase kinase (GSK)-3β expression. Pathways such as Akt/β-catenin and Rb signaling are important in downregulating E-cadherin expression and EMT through the activation of the Snail and Slug genes (6). Since β-catenin was also associated with the Wnt (wingless) and E-cadherin signaling pathways, β-catenin dysregulation may be important in carcinosarcoma. The knowledge of the spectrum of these genes is essential for the establishment and maintenance of phenotypic characteristics of uterine carcinosarcoma. Upregulation of nuclear p53 expression, which occurs as an early event in tumorigenesis, was associated with carcinosarcoma (73%), leiomyosarcoma (38%) and ESS (27%), suggesting that p53 protein accumulation was demonstrated more often in carcinosarcoma (7). HER-2 overexpression and amplification were detected in ∼30% of these cases (8). VEGF overexpression indicated poor survival. Carcinosarcoma had higher ERβ expression but lower ERα and PR expression (9). Although ERβ appears to oppose the proliferative activity of ERα, ERβ might exert a stimulatory effect on the uterus. ERβ expression was associated with an increased risk for death. CATs, including melanoma antigen, family A, 4 (MAGE-A4) and NY-ESO-1 (also known as CTAG1B, cancer/testis antigen 1B), have been shown to be overexpressed in certain carcinomas and sarcomas (10). The upregulation of MAGE-A4 and NY-ESO-1 expression was detected in 91 and 45% of the carcinosarcomas, respectively. COX-2 expression in the two compartments was a potential predictive and unfavorable prognostic marker. These specific proteins are potential candidates that may serve as novel prognostic markers of uterine carcinosarcoma.

Somatic mutations have been described at low frequency in the majority of the tyrosine kinase growth factor gene family and their downstream targets [epidermal growth factor receptor (EGFR), CDKN2A, hepatocyte growth factor receptor (MET), v-kit Hardy-Zuckerman 4 feline sarcoma viral oncogene homolog (KIT), v-Ki-ras2 Kirsten rat sarcoma viral oncogene homolog (KRAS), v-raf murine sarcoma viral oncogene homolog B1 (BRAF), phosphatidylinositol-4,5-bisphosphate 3-kinase, catalytic subunit α (PI3KCA), HER-2 and platelet-derived growth factor receptor (PDGFR)], which are considered critical in carcinogenesis. Mutations of these genes have been identified as biomarkers against the treatment of certain carcinomas with small molecule inhibitors or monoclonal antibodies to the specific growth factor receptor. The role of mismatch repair genes in uterine carcinosarcoma is still controversial.

These findings have implications for the mechanism by which overexpression of the EMT- and cell cycle-related genes regulate sarcomagenesis and cell growth. Obtaining a better knowledge of the dynamic behaviors of these networks is of interest for novel treatment strategies. Much information has been gained with regard to the different genetic changes that lead to its development, while the mechanisms still remain to be elucidated. Additional studies are required to understand the process of sarcomagenesis.

### Therapeutic approaches

For all the types of uterine sarcoma, all the operable patients initially underwent surgery. Management usually includes total abdominal hysterectomy and bilateral salpingo-oophorectomy with or without pelvic lymph node dissection. Adjuvant therapy with radiation therapy and/or chemotherapy is recommended depending on the clinical and pathological factors, including tumor stage, histological subtype, grade, lymphovascular invasion and distant metastasis. Adjuvant radiotherapy comprising a combination of external radiotherapy to whole pelvis followed by intravaginal brachytherapy may be recommended to postoperative patients with early stages of carcinosarcoma. Postoperative radiation appears to improve local control.

A critical review of the literature on adjuvant chemotherapy in uterine carcinosarcoma has been described. Patients with higher stage of disease may be considered for ifosfamide plus paclitaxel combination chemotherapy (11). Although the effect of chemotherapy treatment varied among patients, adjunctive ifosfamide and cisplatin chemotherapy may also improve progression-free survival. Nevertheless, the incidence of tumor recurrence was high.

Therefore, many investigators have actively examined alternative approaches or novel treatment strategies to lower the disease burden. Two hundred and ninety-eight randomized control trials including uterine sarcoma have been listed in ClinicalTrials.gov registries (http://clinicaltrials.gov/). Several phase II and III clinical trials have been conducted to determine the safety, side-effect profiles and the impact on survival and the antitumor effects of paclitaxel plus carboplatin vs. ifosfamide plus paclitaxel (ClinicalTrials.gov Identifier: NCT00954174; http://clinicaltrials.gov/ct2/show/NCT00954174?term=uterine+sarcoma&no_unk=Y&rank=11), liposomal doxorubicin and carboplatin (NCT00032162; http://clinical-trials.gov/ct2/show/NCT00032162?term=uterine+sarcoma&no_unk=Y&rank=13), ifosfamide with or without paclitaxel (NCT00003128; http://clinicaltrials.gov/ct2/show/NCT00003128?term=uterine+sarcoma&no_unk=Y&rank=23), ifosfamide or doxorubicin (NCT00003212; http://clinicaltrials.gov/ct2/show/NCT00003212?term=uterine+sarcoma&no_unk=Y&rank=47) or in patients with uterine sarcomas including carcinosarcoma.

In addition, a phase I randomized study of MAGE-12 peptide vaccine has been conducted in patients with refractory uterine sarcoma expressing MAGE-12 antigen (NCT00020267; http://clinicaltrials.gov/ct2/show/NCT00020267?term=uterine+sarcoma&rank=3). Several phase I, II and III trials have been performed to investigate the best schedule of molecular-targeted therapies and side-effects such as dasatinib [second generation tyrosine kinase inhibitor (TKI)] and ipilimumab (anti-CTLA-4 monoclonal antibody) (NCT01643278; http://clinicaltrials.gov/ct2/show/NCT01643278?term=uterine+sarcoma&rank=7), temsirolimus [mammalian target of rapamycin (mTOR) inhibitor] and vinorelbine ditartrate (NCT01155258; http://clinicaltrials.gov/ct2/show/NCT01155258?term=uterine+sarcoma&rank=10), sunitinib (TKI) (NCT00474994; http://clinicaltrials.gov/ct2/show/NCT00474994?term=uterine+sarcoma&no_unk=Y&rank=31), imatinib mesylate (ABL TKI) (NCT00075400; http://clinicaltrials.gov/ct2/show/NCT00075400?term=uterine+sarcoma&no_unk=Y&rank=66), pazopanib hydrochloride (a small molecule kinase inhibitor that blocks VEGFR, PDGFRα, FGFR1 and c-fms) (NCT01247571; http://clinicaltrials.gov/ct2/show/NCT01247571?term=Uterine+sarcoma&no_unk=Yrank=1), or thalidomide (NCT0000 6005; http://clinicaltrials.gov/ct2/show/NCT00006005?term=uterine+sarcoma&rank=19). However, limited data exist to guide physicians in the treatment of uterine carcinosarcoma.

## 4. Leiomyosarcoma

### Clinical features

Uterine leiomyosarcoma is the second most common subtype of uterine sarcoma, accounting for ∼30% of all cases (1). This disease is prevalent in relatively young women within the age group of 40–55 years. Long-term survivors of hereditary retinoblastoma or prior pelvic radiation have been considered as risk factors. Hormonal conditions such as oral contraceptive users have not been associated with the development of leiomyosarcoma. Leiomyosarcoma has not been associated with hormonal conditions. Most patients had uterus-confined disease presenting at stage I. The probability of ovarian or pelvic lymph node involvement was low.

### Imaging characteristics

MR imaging findings have potential diagnostic significance as they often appear as heterogeneous lesions with a lobulated mass of moderately high-signal intensity on T1-weighted images and high-signal intensity on T2-weighted images (12). Leiomyosarcoma showed a higher incidence of hemorrhage or necrosis compared to carcinosarcoma. Detection of scattered foci of hemorrhage or necrosis offers diagnostic evidence, although this pattern was also observed in degenerating leiomyoma. An additional imaging modality is ^18^F-fluorodeoxyglucose (FDG) positron emission tomography (PET), usually as a hybrid technique in conjunction with computed tomography (CT). ^18^FDG PET/CT and MR imaging are accurate, sensitive and specific modalities for detecting recurrence in post-therapy patients that are able to provide additional independent information.

### Genetic aberrations

Uterine leiomyosarcoma may occur *de novo* or develop within a preexisting leiomyoma. A recent study suggested that uterine leiomyosarcoma occurs in a preexisting leiomyoma (13). To gain insight into the characteristic genes involved in the molecular mechanisms of leiomyosarcoma development, a number of technologies such as genome-wide profiling analyses, computational integration of gene expression profiles, genetics, proteomics and immunohistochemical studies for leiomyosarcoma have been compared to gene expression profiles for leiomyoma or STUMP (14–16). These technologies have quantified the expression of several predicted target genes in leiomyosarcoma. Loss of heterozygosity (LOH), amplification, overexpression and mutations may contribute to the risk of leiomyosarcoma development (Tables I and II). LOH on chromosome 10 occurred more frequently in leiomyosarcoma compared to leiomyoma and may contribute to sarcomagenesis. Amplification and over-expression were detected in the cyclin-dependent kinase 4 (CDK4), p53 E3 ubiquitin protein ligase homolog (MDM2), GLI-Kruppel family member (GLI1) and tetraspanin 31 (TSPAN31) genes of the 12q13–15 region (17). c-Kit overexpression was detected in 53% of cases. Mutation of p53 was observed in 24% of leiomyosarcoma cases.

A significant difference was observed in p16, p21 and p53 expression between leiomyosarcoma and leiomyoma, supporting the hypothesis that these genes may be the predicted target genes that are commonly enriched in sarcoma genesis pathways (Table III). The expression of Ki-67, p53 and p16 was substantially higher in leiomyosarcoma and undifferentiated endometrial sarcoma compared to ESS (18). By contrast, the expression of several tumor suppressor genes [comprising death-associated protein (DAP), phosphatase and tensin homolog (PTEN) and Ras association (RalGDS/AF-6) domain family member 1 (RASSF1A)] was downregulated in leiomyosarcoma specimens compared to leiomyoma. The downregulation was attributed to promoter hypermethylation of these genes. A strong expression of p53, p16, Ki-67, twist homolog 1 (TWIST1), fascin homolog 1 (FSCN1; actin-bundling protein), ESR1 (estrogen receptor 1), PGR (progesterone receptor), RB1, CDKN2A, cyclin D1 (CCND1), CCND3 and phospholipase D1 (PLD1), as well as a low expression of BCL-2 may be associated with poor prognosis (18–20). The TWIST1 gene has a key role in transcriptional regulation in mesenchymal cell lineages. This protein is expressed at high levels in cells undergoing malignant transformation and is associated with tumor progression and metastasis. Fascin, an actin-binding protein, plays a critical role in cell migration, motility and adhesion. Fascin overexpression was associated with the metastasis of various types of cancer by increasing cell motility. The activation of the mTOR-PLD1 pathway in leiomyosarcoma, but not in STUMP, provides insight into their tumorigenic mechanisms (21). Differences in the target gene or protein expression could have implications for the prediction of survival (Table I). These findings identify the cell cycle regulators and their downstream targets as deregulated genes driving multiple sarcomagenic phenotypes in leiomyosarcoma.

**Figure 1:**
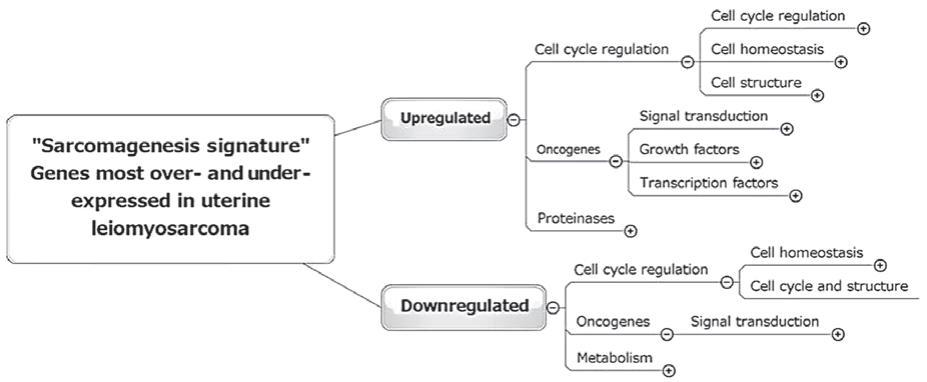
Network analysis of key genes involved in leiomyosarcoma development. The pathway analysis groups the differentially expressed genes into biological mechanisms that are related to leiomyosarcoma development. The gene expression profile of leiomyosarcoma was significantly different from usual leiomyoma. These results demonstrated several significant networks. Of these networks, cell cycle regulation and the oncogene pathway were the most critical.

A comprehensive analysis of differentially regulated genetic signaling pathways was used to select and annotate genes by function and pathway (gene ontology). The most representative biological functions were involved in cell cycle regulation and oncogene-dependent signaling, which undergo a functional change towards mesenchymal cell proliferation during sarcomagenesis (Fig. 1 and Table II). This ‘sarcomagenesis signature’ was paralleled by the necessity of downregulation of putative tumor suppressor genes and mesenchymal cell proliferation. This signature may promote a favorable environment for leiomyosarcoma progression. These analyses may provide insight into the biology of leiomyosarcoma and provide a novel target for molecular-directed therapy.

The mouse model also provides an *in vivo* tool to initiate tumorigenesis and investigate novel therapies. Several investigators (14) reported novel mouse models of uterine smooth muscle malignant tumors in which the tumors exhibit certain features of human leiomyosarcoma. Tumors developed as a result of a germline or somatic mutation in BRCA1, the mediator complex subunit 12 (MED12), low molecular mass polypeptide 2 (LMP2, also known as a proteasome β1i subunit) and tuberous sclerosis 2 (TSC2) genes (Table I). Since BRCA1, a well-known tumor suppressor gene, plays a role in maintaining genomic stability, loss of BRCA1 function potentially is involved in the progression of leiomyosarcoma (22). Frequent mutations of the MED12 gene were identified in 70% of leiomyosarcoma. MED12 is a tumor suppressor gene whose subcomplex includes CDK8 and cyclin C. Therefore, MED12 dysfunction could lead to abnormal cell growth. TSC2 (16p13.3) inhibits mTOR protein and regulates cell growth. Loss of TSC2 is a key event in many types of human cancer: germline inactivating mutations of TSC2-affected patients have an increased risk of developing renal cell carcinoma. LMP2, an interferon (IFN)-γ-inducible factor, was associated with cell cycle regulation. Mice with homozygous LMP2 deletion spontaneously developed uterine leiomyosarcoma (23). Loss of LMP2 expression is a potentially important risk factor for human leiomyosarcoma development. Taken together, abnormalities in several signaling pathways in leiomyosarcoma were identified. The major dysfunctions of differentially expressed pathways included cell cycle regulation.

### Therapeutic approaches

The effective and relatively well-tolerated combination chemotherapy regimens for leiomyosarcoma include doxorubicin plus ifosfamide and gemcitabine plus docetaxel (24). Thus, investigations regarding the efficacy and toxicity of combination chemotherapies should be conducted. Trabectedin (ET-743, Yondelis^®^, Pharma Mar, Madrid, Spain) a synthetized molecule, is a marine-derived antineoplastic agent that disrupts the cell cycle. Trabectedin has been shown to be efficient in phase II trials for leiomyosarcoma. Although disseminated leiomyosarcoma patients have limited chemotherapeutic options, aromatase inhibitors, megestrol and medroxyprogesterone have been used in the treatment of selected patients.

Molecular-targeted therapies have been conducted to assess the efficacy of pazopanib, mTOR, COX-2 and angiogenesis inhibitors. However, clinical trials with imatinib and sunitinib have failed to show improved outcomes as second- or third-line uterine leiomyosarcoma treatment (25). Temsirolimus, an mTOR inhibitor, has been shown to have limited clinical activity (25). A randomized, double-blinded, placebo-controlled phase III trial was conducted to investigate the efficacy and toxicity of pazopanib on progression-free survival in patients with metastatic sarcoma following failure of standard chemotherapy (26). Pazopanib has been reported to have consistent prolongation of progression-free survival (4.6 vs. 1.6 months) and might be a potent oral angiogenesis inhibitor with clinical antitumor activity in a variety of solid tumors.

## 5. Smooth muscle tumor of uncertain malignant potential (STUMP)

### Clinical features

Smooth muscle tumors can be classified as benign, intermediate and malignant. A histopathologic diagnosis of leiomyoma and leiomyosarcoma remains a challenge and depends on histologic criteria such as coagulative tumor cell necrosis, nuclear atypia and mitotic activity. The intermediate category encompasses entities such as STUMP, atypical leiomyomas, or atypical leiomyomas with low malignant potential. STUMPs are uncommon and are atypical smooth muscle tumors. The STUMP category excludes the benign diagnosis of leiomyoma, while the current criteria for leiomyosarcoma are not met. Given the borderline nature of this histopathologic diagnosis, the STUMP category is a heterogeneous group. These tumors cannot be classified as definitively benign or malignant. Therefore, a small number of these tumors constitute a diagnostic dilemma. Their natural history, clinical outcome, optimal management and follow-up measures remain sparse due to the lack of accepted diagnostic criteria.

The mean age at diagnosis was 45 years and 80% of the patients were premenopausal (1). The recurrence rate in patients with uterine STUMP was ∼7%. Consequently, we reconsidered the classification within the intermediate category. With advances in molecular biology and genomic technologies, specific markers were identified as potential diagnosticators of the intermediate category including the STUMP entity (as mentioned below).

### Imaging characteristics

Use of MR imaging may be difficult to accurately and reliably distinguish STUMP from leiomyoma or leiomyosarcoma. Generally, STUMP showed homogeneously low signal on T2-weighted images, which may also occur in leiomyoma. STUMP and leiomyosarcoma shared a number of common MR imaging features: they often present with areas of heterogeneous high T2-signal intensity, indicating that differentiation between these entities is difficult.

### Genetic aberrations

Immunohistochemical study using a well-characterized panel of antibodies to p16, p53 and Ki-67 could pose a challenge, while it may be helpful in distinguishing STUMP from usual leiomyoma and leiomyosarcoma cases (Tables I and III). The Ki-67 and p53 expression of leiomyosarcoma were significantly higher compared to STUMP (27). No significant difference was observed in p16 and p21 levels between leiomyomas, atypical leiomyomas (leiomyoma with bizarre nuclei) and STUMP (28). The expression patterns of these markers showed no significant differences and also overlapped between leiomyosarcoma and bizzare leiomyoma (29). A subset of STUMP characterized by strong, diffuse p16 positivity contains the morphological changes of coagulative tumor cell necrosis (30). These STUMP patients may be classified as leiomyosarcoma. It may be possible to discern meaningful subsets of the STUMP patients with a malignant potential by using p16, p21 and p53 immunostaining. p16 is a cyclin-dependent kinase inhibitor (CDKI) that reduces cell cycle progression. p21 mediates the p53-dependent cell cycle G1 phase arrest and might play an important role in sarcomagenesis. Although limited, p16, p21 and p53 may serve as useful markers in the assessment of problematic uterine smooth muscle tumors.

## 6. Endometrial stromal sarcoma (ESS)

### Clinical features

Endometrial stromal tumors are currently classified into endometrial stromal nodules, ESS and undifferentiated endometrial or uterine sarcoma. ESS constitutes 10–15% of uterine mesenchymal tumors and affects relatively younger women, generally involving patients aged 40 to 55 years (1). Although the majority of malignant tumors arising in ovarian endometriosis are carcinomas, ESS arising in extra-ovarian endometriosis is rarely described. Although the role of female hormones in the etiology of ESS remains speculative, an association with exposure to tamoxifen, unopposed estrogen use and conditions such as polycystic ovaries has been observed. This neoplasm may also develop following radiation treatment.

### Imaging characteristics

ESS resembles adenomyosis, uterine or degenerated leiomyoma on ultrasound imaging. Tumors contained multiple polypoid areas with low signal intensity on T1-weighted images and heterogeneously high-signal intensity on T2-weighted images. Bands of low T2-signal intensity within the myometrium could be observed in ESS patients with myometrial invasion (31). Gadolinium enhancement was mild, although it was present in the solid components.

### Treatment strategies

ESSs are hormone receptor-positive. Although no randomized clinical trials have been conducted to determine the optimal treatment regimen, endocrine therapy using progestin and aromatase inhibitors is a widely used treatment strategy for this disease.

### Genetic aberrations

Aggressive tumor features were associated with changes in the immunohistochemical expression pattern of specific gene products (Tables I and IV). Immunohistochemical analysis showed that leiomyoma and ESS tissues exhibited positive staining for ER and PR. Positive staining for CD10 was observed in only ∼20% of cellular leiomyoma and in any leiomyosarcoma case, while it was evident in 100% of ESS cases (32). CD10 was the most specific marker of ESS (Table IV). Desmin and h-caldesmon were negative in ESS and positive in usual and cellular leiomyoma cases. Nuclear β-catenin expression has been shown to be predominantly positive in ESS, although it was decreased in normal endometrial stroma and undifferentiated endometrial sarcomas (32). ESS appears to be associated with the over-expression of CD10 and β-catenin and a reduced expression of desmin. Thus, the detection of a panel of CD10, desmin, h-caldesmon and β-catenin has been proposed as a possible molecular target for differential diagnosis. In addition, Ki-67, p53 and p16 levels were lower in ESS compared to leiomyosarcoma and undifferentiated endometrial sarcoma cases (18).

Recent genome-wide studies (3–10) have identified complex chromosomal rearrangements as a sarcomagenic mechanism (Table I). A specific translocation t(7;17) (p15–p21;q12–q21) with JAZF1/JJAZ1 rearrangement fusion genes was present in 80% of low-grade ESS (33,34). ESS also carried another programmed t(1;6)(p34;p21) translocation (35). In addition, the 14-3-3 fusion oncogene rearrangements resulting from t(10;17) fusion transcripts were specific for undifferentiated endometrial stroma (36). This genomic rearrangement comprised 14-3-3ɛ (YWHAE) and FAM22 family members (FAM22A or FAM22B). Recent identification of unique genetic fusion may provide novel insights into the understanding of ESS pathogenesis.

## 7. Adenosarcoma

### Clinical features

The age at diagnosis of uterine adenosarcoma is usually after menopause, with a median age of 58 years (1). Patients with adenosarcoma tended to be younger compared to women with carcinosarcoma, although older compared to those with leiomyosarcoma and ESS. Adenosarcoma typically presented as a well-demarcated large polypoid mass, thereby occupying the endometrial cavity, and sometimes protruding into the vaginal cavity. Adenosarcoma has been associated with endometriosis, tamoxifen use or long-term oral contraceptive use. The tumor was composed of a benign epithelial glandular tissue in combination with malignant stromal components. Patients with typical adenosarcoma were more likely to have low-grade and early-stage tumors, a lack of myometrial invasion and metastasis, and carried a favorable prognosis. An adverse prognostic factor was sarcomatous overgrowth, characterized by growth of a high-grade sarcoma comprising >1/4 of the tumor mass. This entity was an aggressive rare variant, associated with poor prognosis (37).

### Imaging characteristics

Adenosarcoma presents as a large, multiseptated cystic mass with a number of heterogeneous polypoid components and might mimic the appearance of gestational trophoblastic disease or carcinosarcoma (38). MR imaging showed low-signal intensity on T1-weighted images and high-singal intensity on T2-weighted images. Areas of high-signal intensity on T1-weighted and T2-weighted images may represent hemorrhage or necrosis, respectively.

### Genetic aberrations

The genetic and epigenetic aberrations remain largely unknown. Immunohistochemical analysis of adenosarcoma without sarcomatous overgrowth showed expression of CD10, WT1, ER, PR, androgen receptor (AR), cytokeratin and muscle markers. This immunophenotype closely resembled that of endometrial stromal tumors (Table I). CD10 was positive for adenosarcoma and endome-trial stromal tumors (39). By contrast, adenosarcoma with sarcomatous overgrowth had a WT1 (+), Ki-67 (+) and p53 (+) immunophenotype. CD10, ER and PR were negative. WT1, a tumor suppressor gene, was positive for adenosarcoma with or without sarcomatous overgrowth. WT1 inhibited apoptosis via p53 and BCL-2 and was highly expressed in cancer cells. CD10 and WT1 may be crucial for the diagnosis of adenosarcomas.

In addition, HMGA1a transgenic mice developed aggressive uterine tumors resembling human uterine adenosarcoma (40). Elevated expression of HMGA1a was associated with a highly malignant phenotype in human tumors. HMGA1a is responsible for carcinogenesis, tumor development, invasion and metastasis possibly through the upregulation of COX-2 expression. COX-2 was also highly expressed in uterine sarcoma cells. However, the molecular mechanisms of sarcomagenesis in adenosarcoma remain to be clarified.

## 8. Conclusion

In this study, we summarized the clinical features, imaging characteristics, genomic and genetic aberrations as well as therapeutic approaches in uterine sarcoma, through a systematic review of the literature. The aim of the present review was to identify targets of frequent genomic and genetic aberrations in uterine sarcomas. Consequently, diagnostically and therapeutically relevant preclinical rodent models for characterizing aberrant oncogenic functions were demonstrated. Fundamental issues associated with the natural history and pathogenesis of uterine sarcomas remain largely unknown.

Uterine carcinosarcoma was a biphasic malignant tumor consisting of epithelial and mesenchymal components arising from a common epithelial cell or stem cell in the uterine endometrium. Genome-wide gene expression profiling, proteomics and immunohistochemical analysis showed that the expression levels of TGF-β, p16, p53, ERβ, CATs, VEGF, HER-2 and PTEN were upregulated (Table I). Hormone receptors (ERα and PR), β-catenin and cyclin D1 were infrequently expressed. Little is known regarding the involvement in tumorigenesis, although TGF-β may be an important mechanistic link between the induction of EMT and sarcomagenesis. In addition, p16 and p53 may have an oncogenic function due to the promotion of cell cycle regulation. These findings suggest that changes in the TGF-β pathway as well as alterations in cell cycle regulation may be essential for the establishment and maintenance of the phenotypic characteristics of uterine carcinosarcoma.

A diagnosis of uterine leiomyosarcoma is performed when 2/3 morphological criteria are met, such as coagulative tumor cell necrosis, diffuse cytological atypia, and mitotic index >10 MFs/10 HPFs. However, distinguishing leiomyosarcoma from certain variants of benign leiomyoma is often difficult due to limitations of the diagnostic criteria and classifications. Ki-67, p53, p16 and p21 were strongly expressed in leiomyosarcoma and a moderate expression of PTEN, FSCN1, ER, PR and MIB1 was also present (Tables I–III). Clinicopathological features including clinical outcome appear to correlate with immunohistochemical profiles. In experimental knockout models, the deficiency or germline mutation in the target genes such as LMP2 caused alterations in the expression of various genes associated with leiomyosarcomagenesis. p16 and p53 pathway abnormalities were associated with cell cycle regulation. The overexpression of cell cycle-related genes may be correlated with sarcomagenesis and progression in leiomyosarcoma. Therefore, mutations or polymorphisms affecting the cell cycle regulation pathways may predispose uterine mesenchymal cells to transformation.

A category of STUMP is used when uterine smooth muscle tumors cannot be histologically diagnosed as definitively benign or malignant. STUMP are a heterogeneous group. The classification of uterine mesenchymal tumors remains a challenge, with considerable overlap regarding terminologies such as STUMP, atypical leiomyoma, atypical leiomyoma with low risk of recurrence, and atypical leiomyoma with low malignant potential. Limited data are available regarding whether there was a significant difference in the expression and localization of p16, p21, p53 and Ki-67 between leiomyosarcoma and STUMP, as well as STUMP and usual leiomyoma (Tables I and III). The altered expression of p16 and p21 genes may demonstrate proliferative and survival advantages of the transformed premalignant leiomyoma cells. Future genome-wide investigation may therefore provide a molecular basis for the tumor biology and terminology for STUMP.

Specific cytogenetic aberrations including chromosomal rearrangements have been identified in endometrial stromal tumors (Tables I and IV). Fragile sites in specific chromosomal regions are prone to breakage. Chromosomal rearrangements that encode a tumor-specific chimeric oncogenic fusion protein appear to be critical events in the carcinogenesis of certain leukemias and solid carcinomas. Previous genome-wide studies have identified complex chromosomal rearrangements in ESS, including JAZF1 (low-grade morphology) and 14-3-3 (YWHAE, undifferentiated morphology), as an oncogenic mechanism. The existence of specific chromosomal rearrangements provides insights into their molecular biology, potential diagnostic markers and possible therapeutic targets.

The HMGA1a gene induced aggressive lymphoid tumors and uterine adenosarcoma in transgenic mice, possibly through the upregulation of inflammatory pathway-related genes such as COX-2 expression (40) (Table I). Future studies should investigate the relationship between HMGA1a expression and adenosarcoma development as well as their clinical perspective in the development of novel therapeutic strategies. Clinicopathological features including morphological criteria and clinical outcome appeared to correlate with immunohistochemical profiles, including Ki-67, p53, WT1 and CD10. The identification of genetic and expression profiles would accelerate the search for diagnostic markers and targets for individualized therapies.

In the present study, we also concisely reviewed the standard chemotherapeutic regimens and additional modalities incorporating peptide vaccine and molecular-targeted therapies including dasatinib, ipilimumab, temsirolimus, vinorelbine ditartrate, sunitinib, imatinib, pazopanib and thalidomide. No effective treatment recommendations and decision algorithms have been found to achieve a high rate of cure or prolong survival, due to the relatively rare incidence of uterine sarcomas, their diversity of histologic types and lack of perception of long-term safety profiles.

In conclusion, this review provides an overview of uterine sarcomas with a focus on the genetic aberrations and genomic rearrangements that are important in uterine sarcomagenesis. The different profile pattern involved in cell cycle regulation might be associated with the pathogenesis of uterine sarcoma. The ultimate goal is to improve the understanding of disease biology and develop more effective therapies.

## Figures and Tables

**Table I:** Review of the clinical features, imaging characteristics and genetic aberrations on uterine sarcoma biology.

Variables	Carcinosarcoma	Adenosarcoma		Leiomyosarcoma		STUMP	Endometrial stromal sarcoma
Gene/protein expression		Without SO	With SO	Better prognosis	Poor prognosis		
Overexpression	TGF-β, p16, p53, ERβ, CATs, VEGF	CD10	Ki-67, p53	BCL-2	Ki-67, p53, p16, p21	p16^a^	CD10, β-catenin
Moderate expression	HER-2, PTEN	WT1	WT1		PTEN, FSCN1, ER, PR, MIB1	FSCN1, p16, p21	Ki-67, p53, p16
Low expression or absence	ERα, PR, IGF1R, CD10		CD10, ER, PR	Ki-67, p53, p16, TWIST1	BRCA1, MED12	Ki-67, p53, MIB1	
Specific genetic aberrations	Chromosomal amplification 8q (c-myc), 20q (ZNF217)	HMGA1a		LOH on chromosome 10, LMP2 deficiency			JAZF (LGESS), 14-3-3 (undiff. ES)
Age	Between the 5th and 7th decades of life, with a mean age of 65 years	The mean age at presentation was 58 years		Relatively young women in the age group of 40–55 years		The mean age was 45 years and 80% were premenopausal	The mean age at presentation was 40–55 years
Clinical manifestations	Vaginal bleeding, enlarged uterus and post-menopausal bleeding	Abnormal vaginal bleeding		Most patients had uterus-confined disease		The recurrence rate was 7%	Abnormal vaginal bleeding and pain
Gross examination	Typically resembled a bulky polypoid papillary mass	A well-demarcated polypoid mass that developed in the endometrium and protruded through the cervical os		Higher incidence of hemorrhage or necrosis compared to carcinosarcoma		Difficult to distinguish STUMP from leiomyoma or leiomyosarcoma	Soft, tan and polypoid may fill the endometrial cavity
Magnetic resonance imaging	Heterogeneous high intensity on T2-weighted MR images and low intensity on T1-weighted images, as well as heterogeneous enhancement of the tumor showing intense enhancement	Large, multiseptated cystic mass with multiple heterogeneous solid components with low-signal intensity on T1-weighted images and high-signal intensity on T2-weighted images		Lobulated mass of moderately high-signal intensity on T1-weighted images and high-signal intensity on T2-weighted images		MR imaging may be difficult to distinguish STUMP from leiomyoma or leiomyosarcoma	Bands of low T2 signal intensity within the myometrial involvement may be observed

a p16 overexpression was characterized by the morphological changes of coagulative tumor cell necrosis. ER, estrogen receptor; PR, progesterone receptor; LOH, loss of heterogeneity.

**Table II:** Genes most over- and underexpressed in uterine leiomyosarcoma.

Functional classification	Abbreviations	Functions of unfamiliar genes
Upregulated		
Cell cycle regulation	CDKN2A (cyclin-dependent kinase inhibitor 2A), CDK4 (cyclin-dependent kinase 4), CDKN2A (cyclin-dependent kinase inhibitor 2A, also known as p16), CCND1 (cyclin D1) and CCND3 (cyclin D3), CKS2 (CDC28 protein kinase regulatory subunit 2), FOXM1 (forkhead box M1), FOXM1 (forkhead box M1), PTTG1 (pituitary tumor-transforming 1), PRC-1 (protein regulator of cytokinesis 1), UBE2C (ubiquitin-conjugating enzyme E2C), COPS3 (COP9 constitutive photomorphogenic homolog subunit 3), MDM2 (p53 E3 ubiquitin protein ligase homolog)	CKS2, binding to the catalytic subunit of cyclin-dependent kinases; FOXM1, a transcriptional activator involved in cell proliferation and regulation of the expression of several cell cycle genes, such as cyclins B1 and D1; PTTG1, an anaphase-promoting complex (APC) substrate with transforming activity; PRC-1, associated with mitotic spindles during mitosis; UBE2C, a gene required for the destruction of mitotic cyclins and for cell cycle progression
Cell homeostasis	GRIA2 (glutamate receptor, ionotropic, AMPA 2), NPTX2 (neuronal pentraxin II), CRABP2 (cellular retinoic acid binding protein 2), POPDC2 (popeye domain containing 2), ST5 (suppression of tumorigenicity 5), TOP2A [topoisomerase (DNA) IIα 170 kDa]	CRABP2, a cytosol-to-nuclear shuttling protein for retinoic acid; POPDC2, regulation of cell-cell adhesion and cell migration; ST5, a regulator of MAPK1/ERK2 kinase, which may contribute to its ability to reduce the tumorigenic phenotype; TOP2A, a gene involved in processes such as chromosome condensation, chromatid separation and relief of torsional stress that occurs during DNA transcription and replication
Cell structure	ACTC1 (actin), DIAPH3 [diaphanous homolog 3 (*Drosophila*)], DCX (doublecortin), COL5A2 (collagen, type V, α2), COPS3 (COP9 constitutive photomorphogenic homolog subunit 3), THBS2 (thrombospondin 2), PLP1 (proteolipid protein 1)	DIAPH3, involved in actin remodeling and regulation of cell movement and adhesion; COPS3, a gene proposed to target p53 protein for proteasome-mediated degradation
Signal transduction	MAP3K8 (mitogen-activated protein kinase 8), PIK3R1 (phosphoinositide-3-kinase, regulatory subunit 1), IL17B (interleukin 17B), TSPAN31 (tetraspanin 31), SPP1 (secreted phosphoprotein 1, also known as Osteopontin)	IL17B, a T cell-derived cytokine that stimulates the release of TNF-α and IL-1β; SPP1, also known as OPN (Osteopontin), an integrin-binding glycophosphoprotein produced by a variety of tissues (the expression of OPN has been associated with poor prognosis in several tumor types)
Growth factors	IGF1 (insulin-like growth factor 1), IGFBP5 (insulin-like growth factor binding protein 5), TGFB3 (transforming growth factor β3)	
Transcription factors	E2F1 (E2F transcription factor 1), RB1 (retinoblastoma 1), GLI1 (GLI family zinc finger 1)	
Proteinases	MMP9 (matrix metallopeptidase 9), CAPN6 (calpain 6)	
Downregulated		
Metabolism	ALDH1A1 (aldehyde dehydrogenase 1 family, member A1), ALDH1B1 (aldehyde dehydrogenase 1 family, member B1)	ALDH1A1, associated with retinol metabolism
Cell cycle and structure	CDKN1A (cyclin-dependent kinase inhibitor 1A (p21, Cip1), DPT (dermatopontin), KRT19 (keratin 19), CNN1 (calponin 1, basic, smooth muscle)	DPT, an extracellular matrix protein with possible functions in cell-matrix interactions and matrix assembly; KRT19, a gene responsible for the structural integrity of epithelial cells; CNN1, a basic actin-binding protein capable of inhibiting smooth muscle contraction, and a constitutive element of smooth muscle cells. Reduced expression of CNN1 in leiomyosarcoma. CNN1 is suspected to have a biological role as a tumor-suppressor; IRF1, interferon regulatory transcription factor and regulation of apoptosis and tumor-suppression; CDKN1A, regulator of cell cycle progression at G1
Cell homeostasis	TNXB (tenascin XB)	TNXB, a member of the tenascin family of extracellular matrix glycoproteins with anti-adhesive effects
Oncogenes	Mutations in KIT (v-kit Hardy-Zuckerman 4 feline sarcoma viral oncogene homolog), MED12 (mediator complex subunit 12), IRF1 (interferon regulatory factor 1)	KIT, mutations are associated with gastrointestinal stromal tumors, mast cell disease, acute myelogenous leukemia and piebaldism; MED12, a transcriptional regulator that binds with a CDK8 subcomplex and functions as a tumor suppressor gene
Signal transduction	MAP3K5 (mitogen-activated protein kinase 5), RNASE4 (ribonuclease, RNase A family, 4)	MAP3K5, an activator of c-Jun N-terminal kinase (JNK)/stress-activated protein kinase (SAPK) and RNASE4, a regulator of mRNA cleavage

Unique genes were classified into several functional gene families: cell cycle regulation, cell homeostasis, cell structure, signal transduction, growth factors, transcription factors, proteinases, metabolism and oncogenes.

**Table III:** Diagnostic immunomarkers for leiomyosarcoma, STUMP and leiomyoma variants.

Genes	Leiomyosarcoma	STUMP	Leiomyoma variants		Usual leiomyoma
			Bizarre	Cellular	
p16	2+ diffuse	1+	1+ to 2+	1+	0 to 1+ focal
p21	2+	1+	1+ to 2+	1+	0 to 1+
p53	2+	1+	1+ to 2+	0	0
Ki-67	1+	1+	0 to 1+	0	0

Immunohistochemical staining techniques were applied to evaluate the frequencies of p16, p21, p53 and Ki-67 expression. 0, absent; 1+, positive; 2+, strongly positive. STUMP, smooth muscle tumor of uncertain malignant potential.

**Table IV:** Status of the immunohistochemical staining profiling of the confirmed proteins, CD10, desmin and β-catenin in patients with ESS.

Proteins	Endometrial stromal nodules	Low-grade ESS	Undifferentiated endometrial sarcomas	Cellular leiomyomata
CD10	2+	1+	0 to 1+	0
Desmin	0 to 1+	0 to 1+	0 to 1+	2+
β-catenin	2+	2+	1+	0

The expression of each protein was investigated by immunohistochemical staining. Due to the low incidence of endometrial stromal sarcoma (ESS), a combination of staining profiling data was obtained from several studies.
